# Classification and risk factors of Post COVID-19 condition: a longitudinal study in the Belgian population

**DOI:** 10.1093/eurpub/ckac129.136

**Published:** 2022-10-25

**Authors:** P Smith

**Affiliations:** Department of Epidemiology and Public Health, Sciensano, Brussels, Belgium

## Abstract

**Background:**

Since the onset of the COVID-19 pandemic, most research has focused on the pathophysiology and management of the acute symptoms of COVID-19, yet some people tend to experience symptoms beyond the acute phase of infection, that is, Post COVID-19 condition (PCC). However, evidence on the prevalence of PCC, its symptoms, and mechanisms are still scarce. This study aimed to assess the distribution, patterns of symptoms, and associated factors of PCC in adult with confirmed COVID-19 infection in Belgium.

**Methods:**

This is a longitudinal cohort study of Belgian adult population with recent COVID-19 infection confirmed via a molecular test and systematically recruited via national tracing call centers. A total of 5181 people were followed-up using online questionnaires at the time of their infection and 3 months later (from April 2021 to February 2022). Their physical, social and mental health was evaluated as well as their acute COVID-19 symptoms and persistent PCC symptoms. These different variables were self-reported.

**Results:**

Half of the participants reported PCC (49.6%). The most frequent persistent symptoms 3 months after infection were fatigue (28%), headache (18%), and memory problems (12%). Women (OR = 1.67,CI95%=1.40-1.99), people with a lower level of education (OR = 1.23,CI95%=1.02-1.48), obese people (OR = 1.23,CI95%=1.02-1.48), people with chronic disease (OR = 1.97,CI95%=1.40-2.77), and people with a higher number of acute COVID-19 symptoms (OR = 2.56, CI95%=1.83-3.58) or hospitalised (OR = 2.19,CI95%=1.25-3.82) were more likely to report PCC. Finally, a latent class analysis on the 29 PCC symptoms highlighted 3 different classes of symptoms.

**Conclusions:**

With the growing number of people infected with COVID-19, PCC is becoming an important public health issue. To allow people with PCC to recover, it is essential to have a multidisciplinary approach and to provide early post-acute physical and psychological rehabilitation interventions according to symptom patterns.

